# Supplementary material for: Metabolomic and Gene Expression Studies Reveal the Diversity, Distribution and Spatial Regulation of the Specialized Metabolism of Yacón (Smallanthus sonchifolius, Asteraceae)
Source: Int J Mol Sci. 2020 Jun 26;21(12):4555. doi: 10.3390/ijms21124555 (PMC7348818; doi:10.3390/ijms21124555)
Supplement: Supplementary file 1 [file ijms-21-04555-s001.zip › Supplementary_Information.docx]

Exploring the specialized metabolite diversity of two yacón (*Smallanthus sonchifolius* (Poepp. & Endl.) H. Robinson) cultivars based on metabolomic and gene expression approaches

Guillermo F. Padilla-González ^1,2,^*, Evelyn Amrehn ^3^, Maximilian Frey ^3^, Javier Gómez-Zeledón ^3^, Alevtina Kaa ^3^, Fernando B. Da Costa ^1^ and Otmar Spring ^3^

^1^ AsterBioChem Research Team, Laboratory of Pharmacognosy, School of Pharmaceutical Sciences of Ribeirão Preto, University of São Paulo, Av do café s/n, 14040-903 Ribeirão Preto, SP, Brazil.

^2^ Jodrell Laboratory, Royal Botanic Gardens, Kew, Kew Green Road, TW9 3AB, London, United Kingdom.

^3^ Department of Biochemistry of Plant Secondary Metabolism, Institute of Biology, University of Hohenheim, Garbenstraße 30, 70599 Stuttgart, BW, Germany.

***** Correspondence: f.padilla@kew.org; Tel.: +44-20-8332-5375

**Supplementary Information**


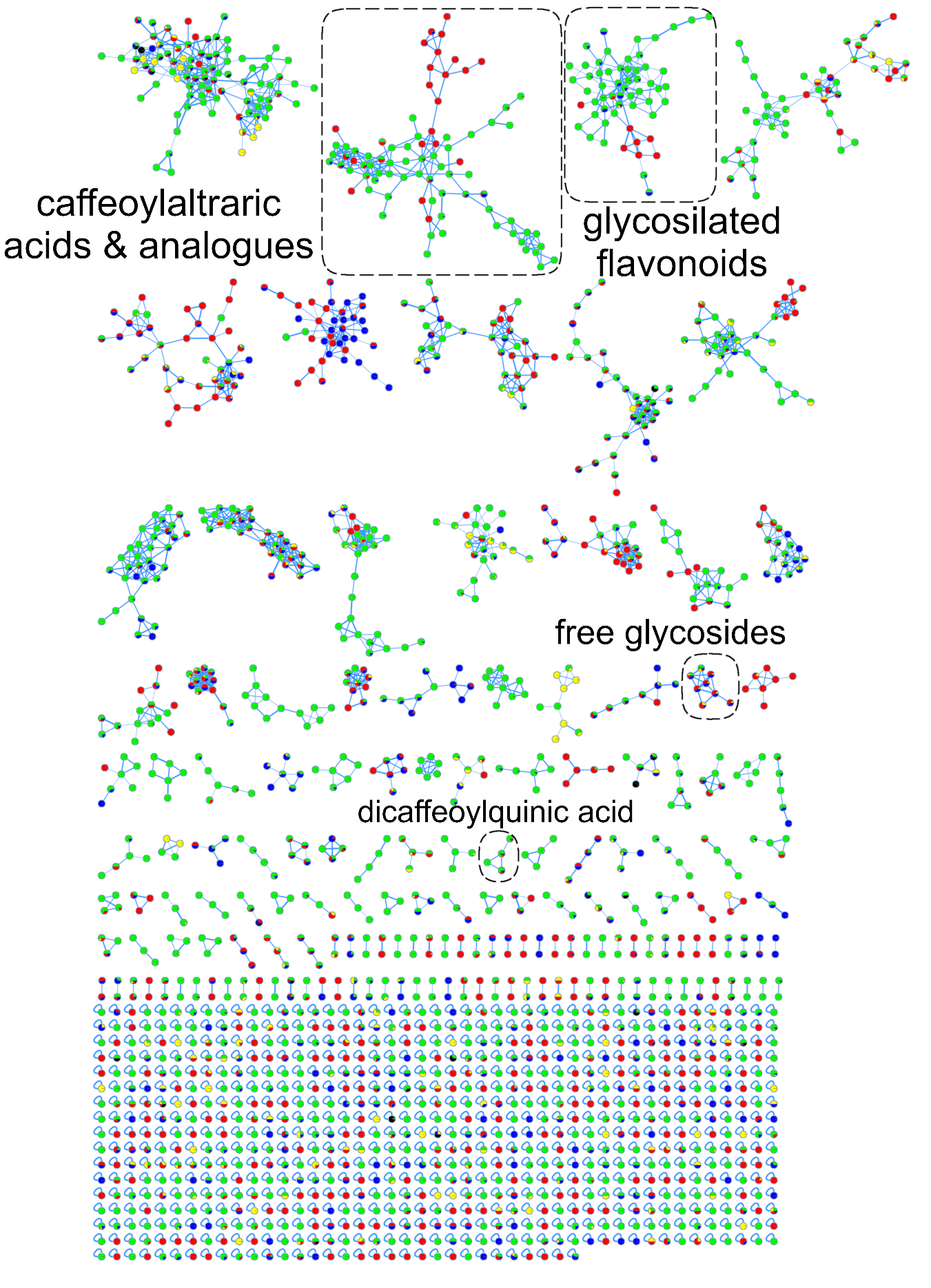


**Figure S1.** Molecular networking of yacón extracts based on metabolic fingerprinting by UHPLC-UV-HRMS/MS in negative ion mode. Node colors represent different organs of yacón (green: leaves, red: roots, blue: stems and yellow: bracts).


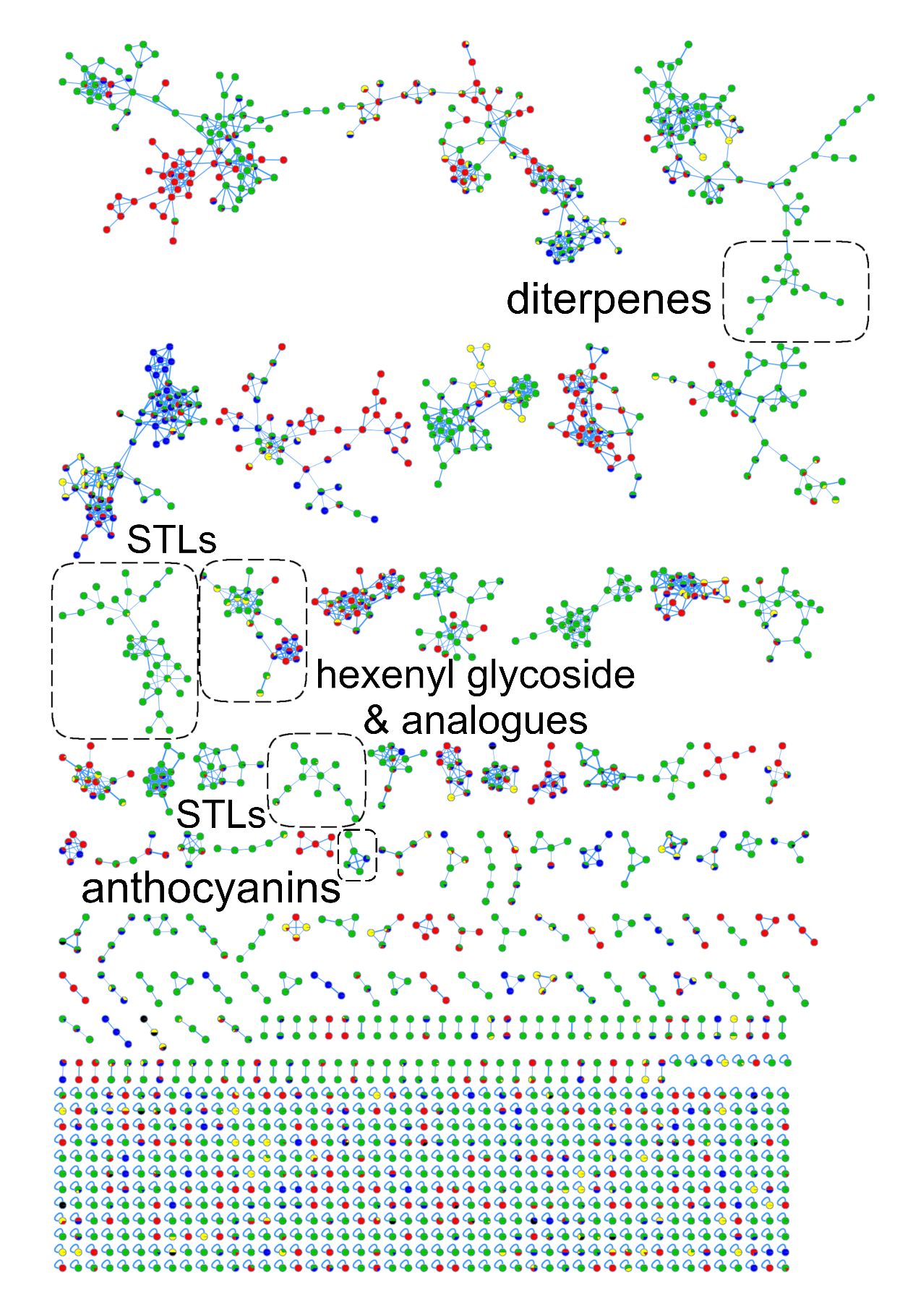


**Figure S2.** Molecular networking of yacón extracts based on metabolic fingerprinting by UHPLC-UV-HRMS/MS in positive ion mode. Node colors represent different organs of yacón (green: leaves, red: roots, blue: stems and yellow: bracts).


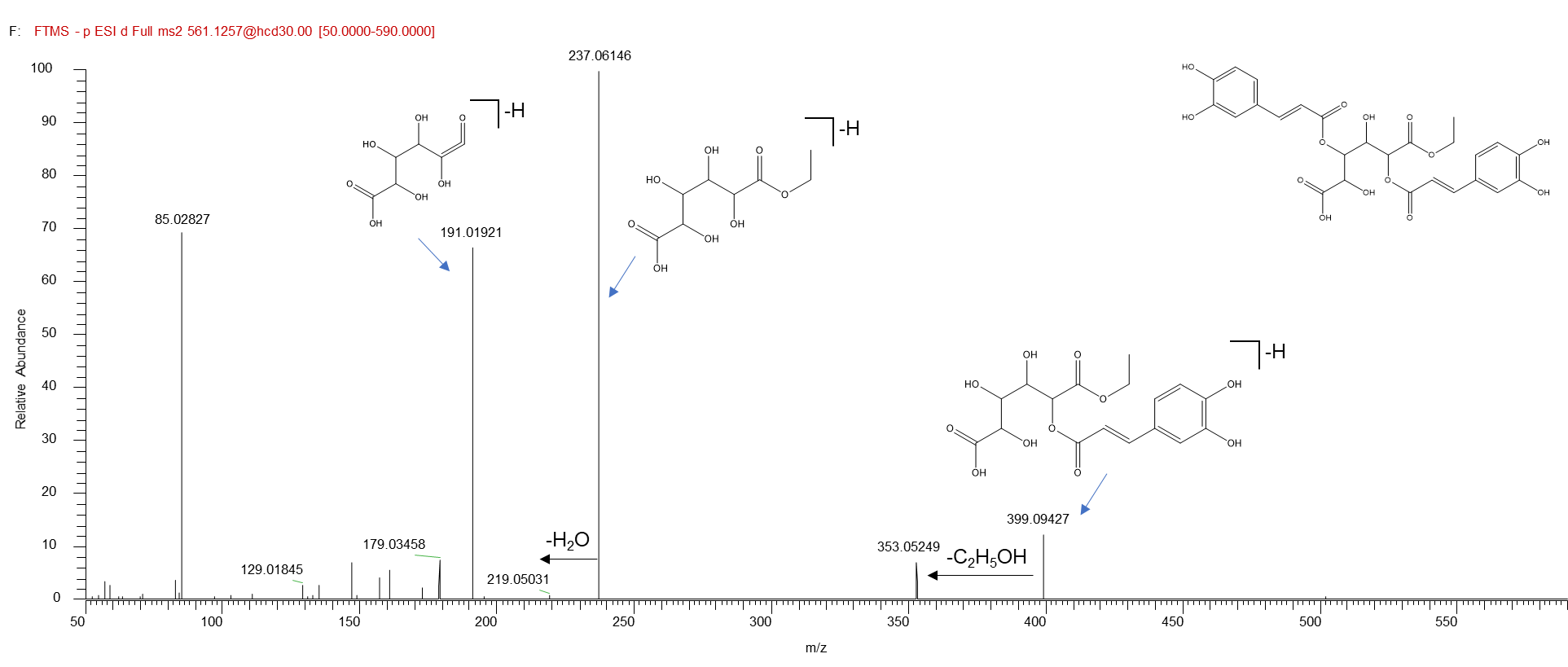


**Figure S3.** MS^2^ spectrum and proposed fragmentation pattern for compound 16, suggested as dicaffeoylaltraric acid monoethyl ester.


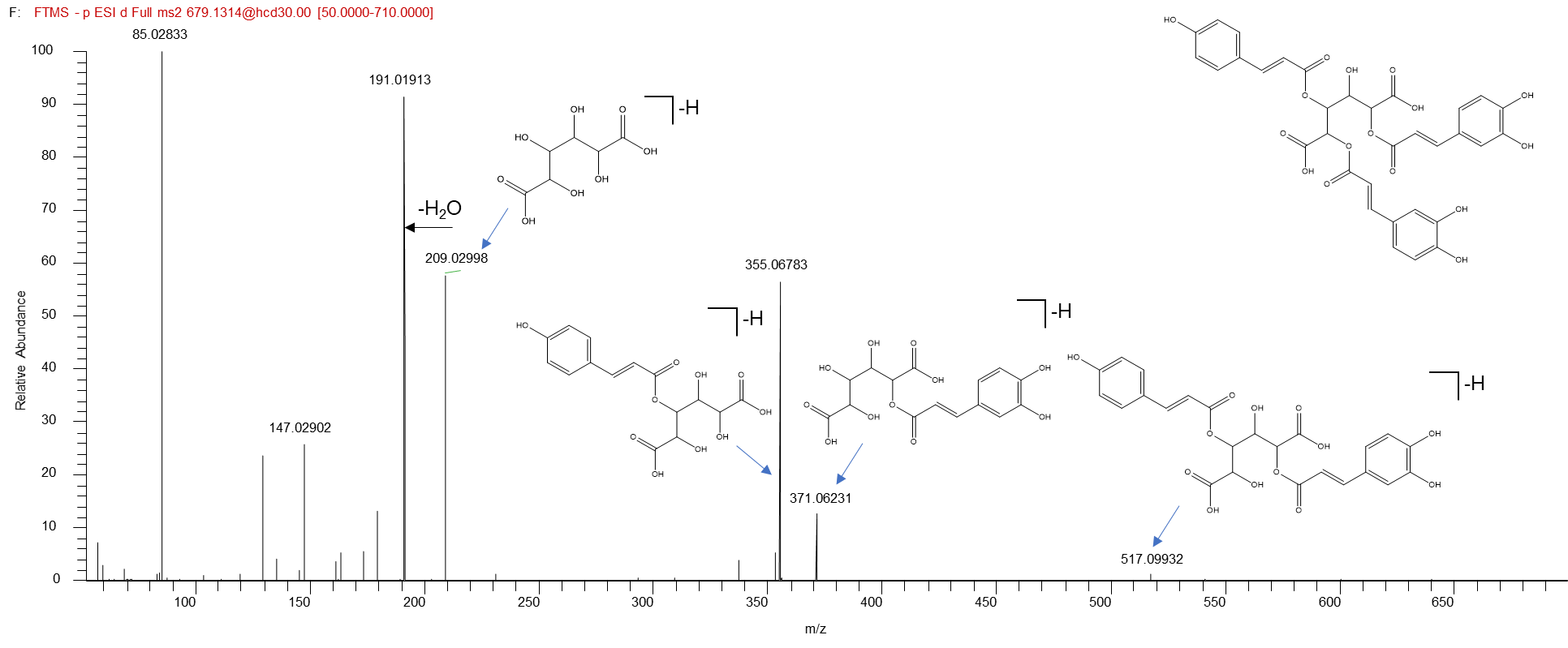


**Figure S4.** MS^2^ spectrum and proposed fragmentation pattern for compound 28, suggested as dicaffeoylcoumaroylaltraric acid.


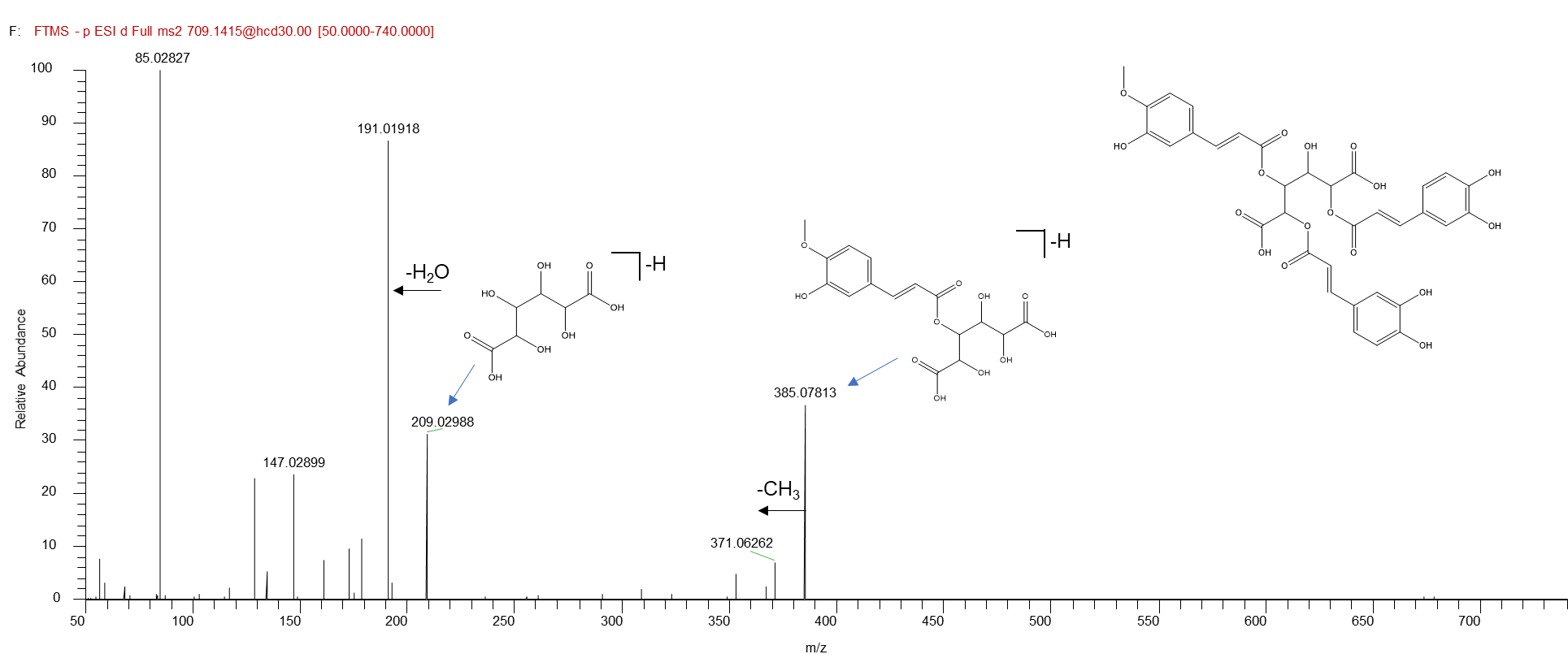


**Figure S5.** MS^2^ spectrum and proposed fragmentation pattern for compound 29, suggested as dicaffeoylferuloylaltraric acid.


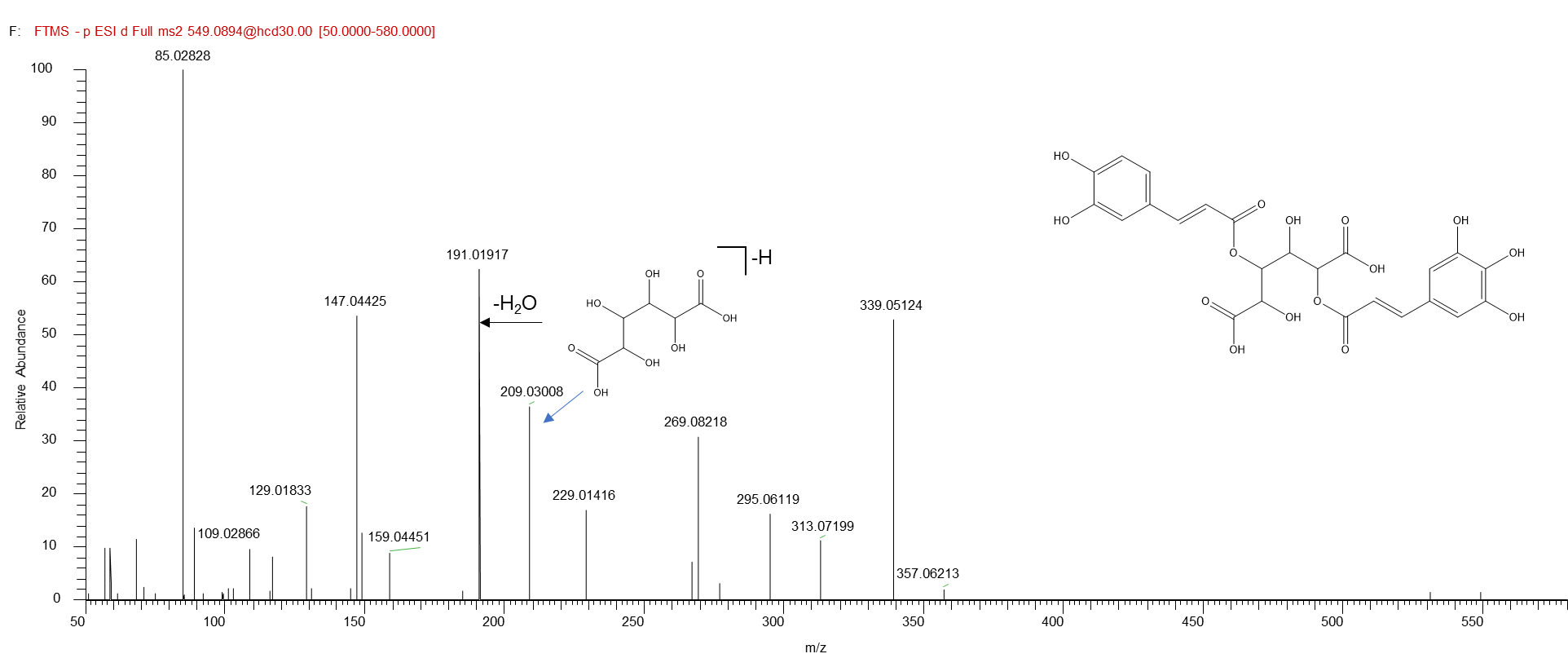


**Figure S6.** MS^2^ spectrum and proposed fragmentation pattern for compound 15, suggested as caffeoyltrihydroxycinnamoylaltraric acid.


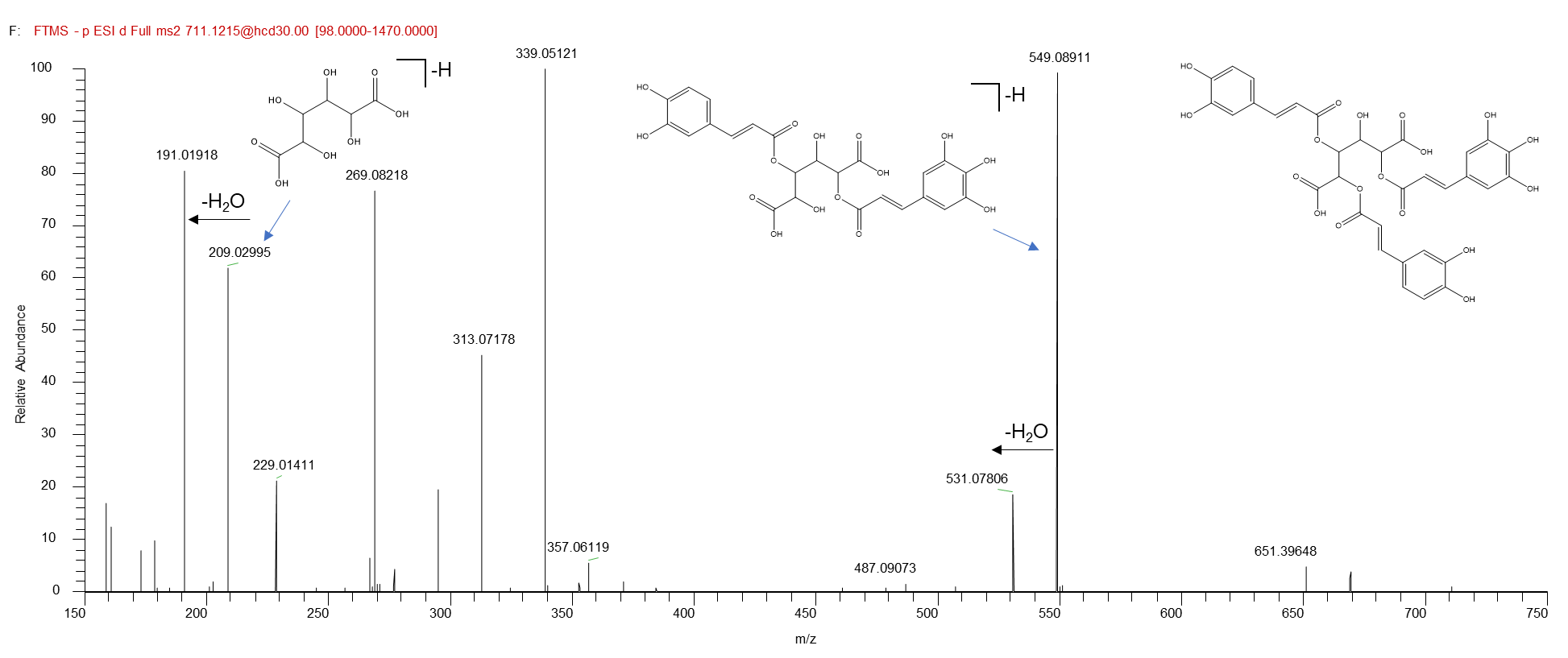


**Figure S7.** MS^2^ spectrum and proposed fragmentation pattern for compound 17, suggested as dicaffeoyltrihydroxycinnamoylaltraric acid.


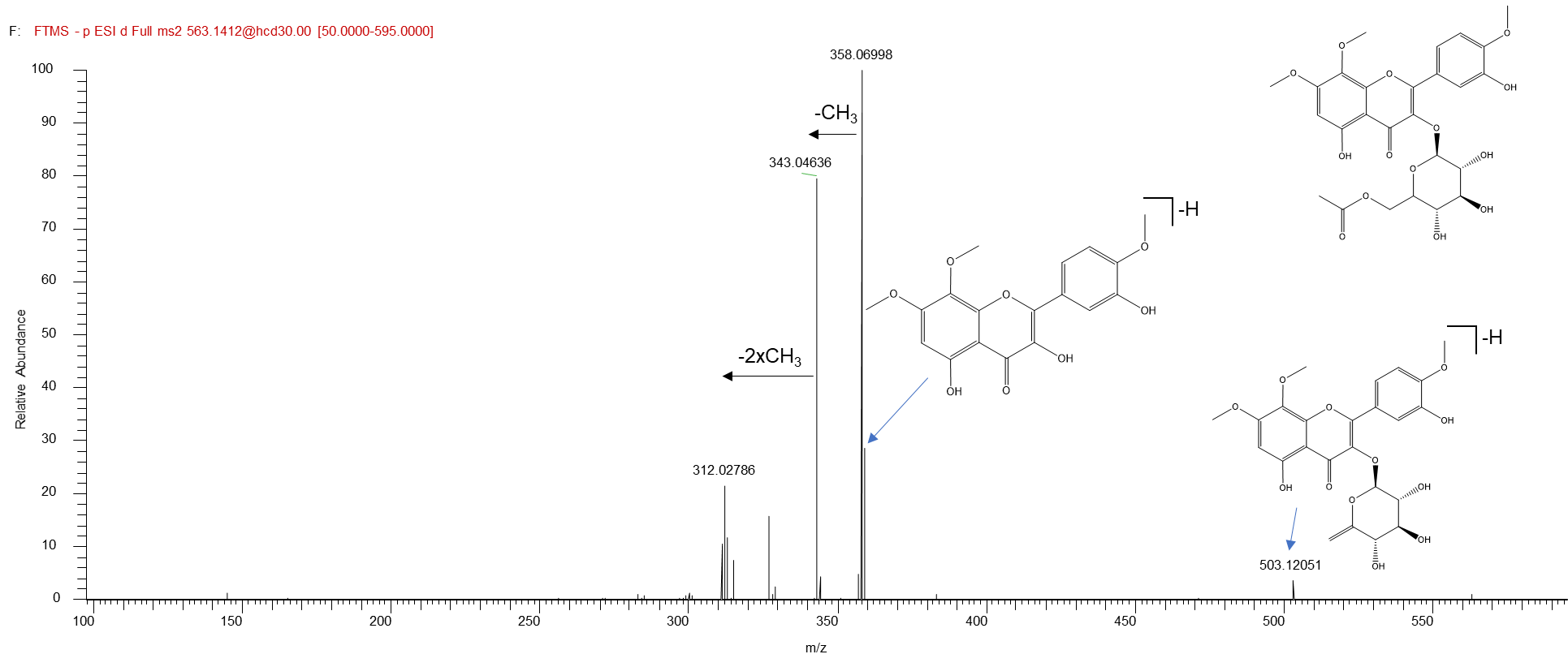


**Figure S8.** MS^2^ spectrum and proposed fragmentation pattern for compound 52, suggested as trimethoxygossypetin-3-*O*-acetylhexoside.


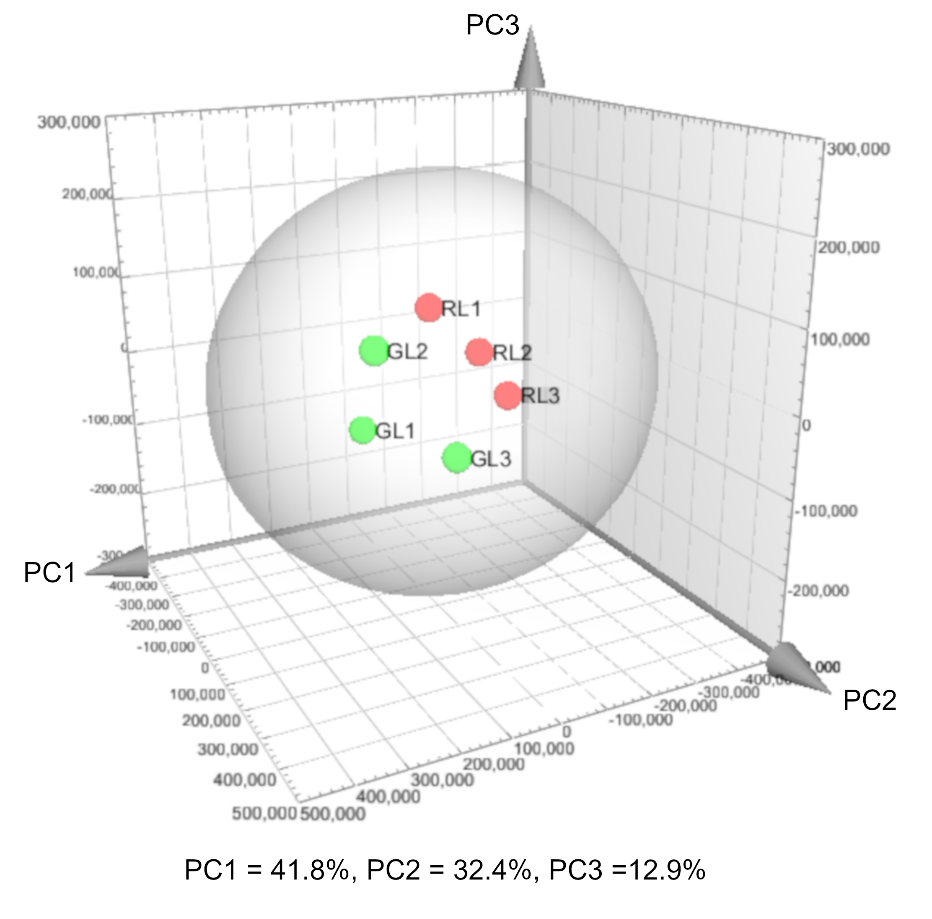


**Figure S9.** OPLS-DA scores plot (R2 = 1.00, Q2 = 0.99) based on metabolic profiling by UHPLC-UV-HRMS in positive ion mode of leaves from two yacón cultivars. Samples colored according to their cultivar (green for the “white cultivar” and red for the “red” cultivar).


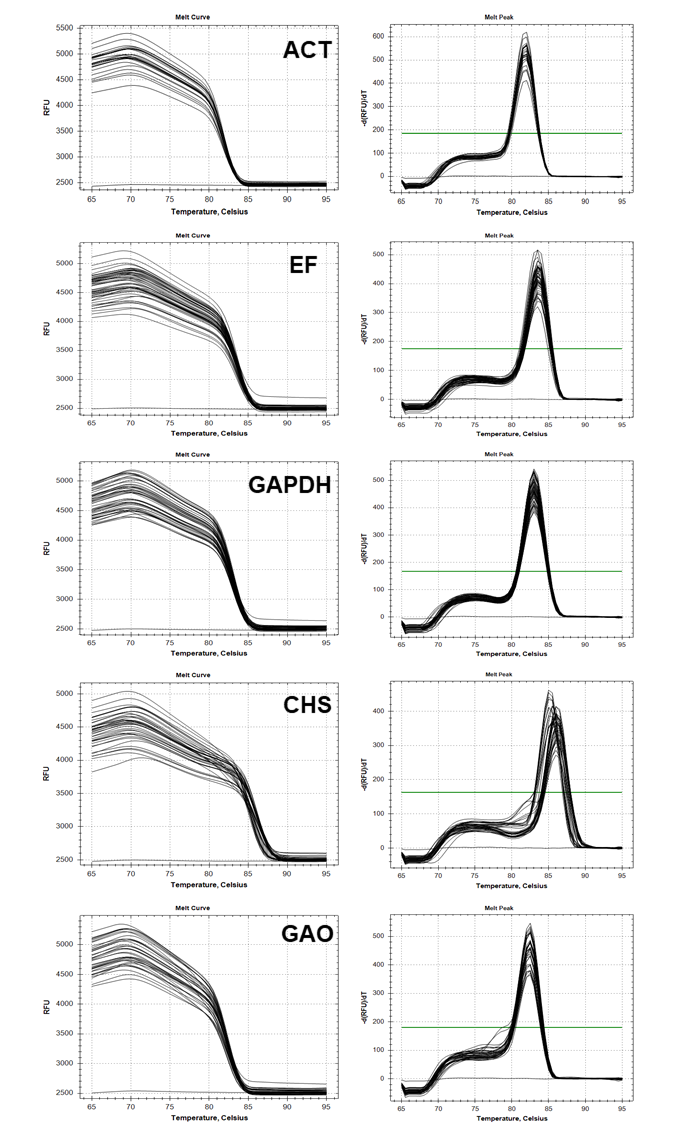


**Figure S10.** Melt curves of all primers used in the RT-qPCR analyses.


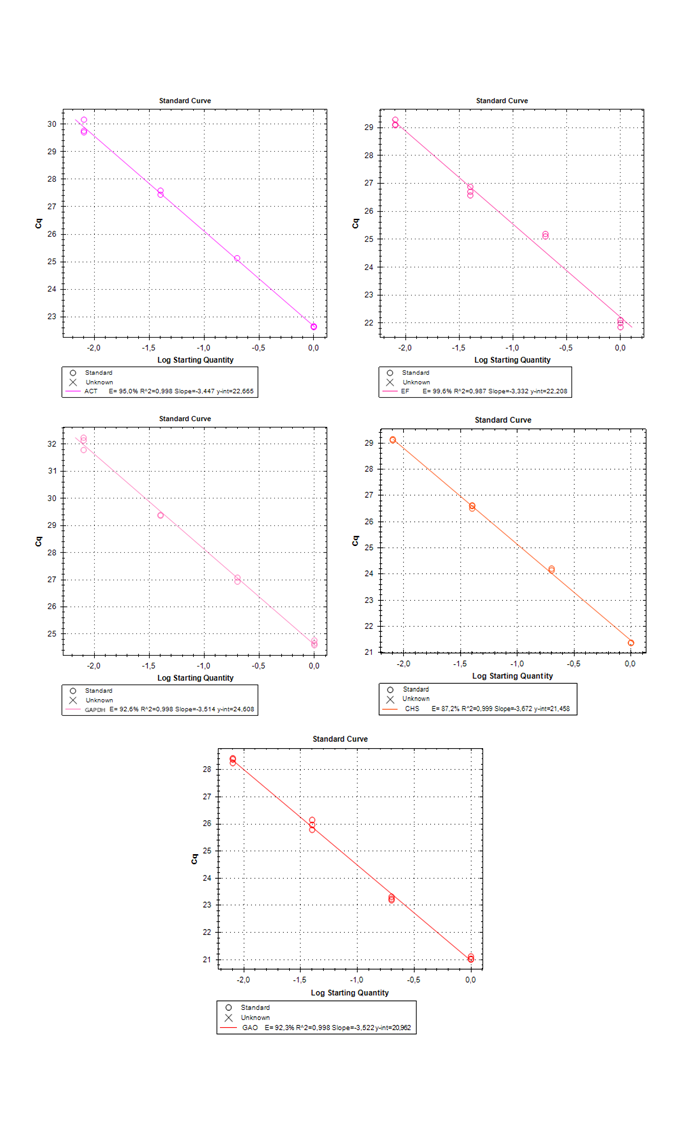


**Figure S11.** q-PCR primers efficiency calculated using four serial dilutions of 1:5.
